# Tumor Cell Expression of Vascular Endothelial Growth Factor Receptor 2 Is an Adverse Prognostic Factor in Patients with Squamous Cell Carcinoma of the Lung

**DOI:** 10.1371/journal.pone.0080292

**Published:** 2013-11-14

**Authors:** Timothy R. Holzer, Angie D. Fulford, Drew M. Nedderman, Tara S. Umberger, Rebecca R. Hozak, Adarsh Joshi, Symantha A. Melemed, Laura E. Benjamin, Gregory D. Plowman, Andrew E. Schade, Bradley L. Ackermann, Robert J. Konrad, Aejaz Nasir

**Affiliations:** 1 Lilly Research Laboratories, Eli Lilly and Company, Indianapolis, Indiana, United States of America; 2 Oncology Statistics-Lilly Research Laboratories, Eli Lilly and Company, Indianapolis, Indiana, United States of America; West German Cancer Center, Germany

## Abstract

A robust immunohistochemical (IHC) assay for VEGFR2 was developed to investigate its utility for patient tailoring in clinical trials. The sensitivity, specificity, and selectivity of the IHC assay were established by siRNA knockdown, immunoblotting, mass spectrometry, and pre-absorption experiments. Characterization of the assay included screening a panel of multiple human cancer tissues and an independent cohort of non-small cell lung carcinoma (NSCLC, n = 118) characterized by TTF-1, p63, CK5/6, and CK7 IHC. VEGFR2 immunoreactivity was interpreted qualitatively (VEGFR2 positive/negative) in blood vessels and by semi-quantitative evaluation using H-scores in tumor cells (0–300). Associations were determined among combinations of VEGFR2 expression in blood vessels and tumor cells, and clinico-pathologic characteristics (age, sex, race, histologic subtype, disease stage) and overall survival using Kaplan-Meier analyses and appropriate statistical models. VEGFR2 expression both in blood vessels and in tumor cells in carcinomas of the lung, cervix, larynx, breast, and others was demonstrated. In the validation cohort, 99/118 (83.9%) NSCLC tissues expressed VEGFR2 in the blood vessels and 46/118 (39.0%) showed high tumor cell positivity (H-score ≥10). Vascular and tumor cell expression were inversely correlated (*p* = 0.0175). High tumor cell expression of VEGFR2 was associated with a 3.7-fold reduction in median overall survival in lung squamous-cell carcinoma (SCC, n = 25, *p* = 0.0134). The inverse correlation between vascular and tumor cell expression of VEGFR2 and the adverse prognosis associated with high VEGFR2 expression in immunohistochemically characterized pulmonary SCC are new findings with potential therapeutic implications. The robustness of this novel IHC assay will support further evaluation of its utility for patient tailoring in clinical trials of antiangiogenic agents.

## Introduction

Vascular endothelial cell growth factor (VEGF) is a potent mitogen and specific mediator of angiogenesis. VEGF signaling is predominately mediated by VEGF receptor-2 (VEGFR2 or kinase insert domain receptor, KDR) [1], [2], which exhibits protein tyrosine kinase activity in response to VEGF ligands [3]. Substantial evidence shows that VEGFR2 is a valid therapeutic target in human cancer. Overexpression of VEGF and VEGFR2 is associated with invasion and metastasis in a number of malignancies [4]. In addition, VEGF/VEGFR2 expression and microvessel density are correlated in several tumor types. This increased microvessel density is associated with poor prognosis in patients with a variety of carcinomas including non-small cell lung cancer (NSCLC) [5]. In preclinical animal models, a murine specific anti-VEGFR2 monoclonal antibody inhibits the growth of human tumor xenografts and causes decreased microvessel density, tumor cell apoptosis, decreased tumor cell proliferation, and extensive tumor necrosis [6], [7]. In early phase clinical studies, a recombinant human monoclonal antibody (IgG_1_) that specifically binds to the extracellular domain of human VEGFR2, ramucirumab (IMC-1121B [LY3009806]), has demonstrated preliminary evidence of efficacy in a variety of human tumors including NSCLC, renal carcinoma, hepatocellular carcinoma, melanoma, ovarian cancer, and colorectal cancer [8]–[13].

Expression of VEGFR2 in vascular endothelial cells is well established. VEGFR2 protein can also be detected within tumor cells of human colorectal [14], [15], breast [16]–[19], and non-small cell lung [20]–[22] cancers among other tumor types [23]–[28]. However, the validity of VEGFR2 expression data in human tumor cells has become a subject of recent interest [29]. A number of factors can be attributed to varied conclusions about the clinical significance of tumor cell expression of VEGFR2 that are reported. These include limited availability of reliable, specific and high-affinity antibodies for archival human tissues, lack of standardization of assay conditions, and a need for optimal quality tissue controls among others. In a recent report, a panel of monoclonal and polyclonal antibodies against VEGFR2 was tested. The authors conclude that only antibody clone 55B11 is specific for VEGFR2 and recommend this clone for use in immunohistochemistry (IHC) [29].

The objectives of this study were to develop a robust VEGFR2 IHC assay using a commercially available anti-VEGFR2 monoclonal antibody (clone 55B11) to demonstrate selective and specific endothelial cell and tumor cell staining on various human cancer tissues, and to evaluate its analytical performance and clinical utility on an independent cohort of NSCLC tissues.

## Materials and Methods

### Ethics Statement

For human tissues obtained from Indivumed (Kensington, MD) for this study, patients sign a written consent form permitting the commercial use of donated tissue. When obtaining and using human tissue, Indivumed acts in strict compliance with The Declaration of Helsinki and The Convention for the Protection of Human Rights and Dignity of the Human Being with regard to the Application of Biology and Medicine: Convention on Human Rights and Biomedicine. Asterand (Detroit, MI) confirms that the following activities have been completed by their collaborators (as necessary) in the process of obtaining human tissues: institutional and independent review board approval, privacy officer authorization, government licenses, or industry accreditations. All informed consent forms used by Asterand are subject to review and approval by appropriate regulatory and ethics authorities. In circumstances where a consent form is unavailable, Asterand obtains a waiver of informed consent from an institutional review board to enable the research use of the tissues and clinical information. Use of human tissues in the NSCLC tissue microarray was approved by the Yale institutional IRB (HIC protocol 9500008219). Consent procedures were approved by the ethics committee, and written informed consent was provided as part of the surgical consent form signed by each patient which included usage of tissue for research.

### Cell culture and RNA interference

The human cell line H441, derived from a patient with papillary adenocarcinoma of the lung, was obtained from ATCC (Manassas, VA). RNA interference was carried out to manipulate VEGFR2 protein levels in H441 with the goal of substantiating the specificity and sensitivity of VEGFR2 IHC. Cells were propogated at 37°C and 5% CO_2_ in RPMI 1640 Medium (Thermo Scientific, Rockford, IL) supplemented with 10% (v/v) fetal bovine serum, 1 M HEPES, 100 mM sodium pyruvate, and 1% (v/v) penicillin-streptomycin. siRNAs included VEGFR2 (KDR, siRNA ID: s7824; Applied Biosystems, Carlsbad, CA) and two control genes: a relevant negative control gene, VEGFR1 (Flt-1, s5289), and a housekeeping gene, GAPDH (4390849, data not shown). Cells at 30–60% confluence were transfected with 1.5 nM siRNAs using Lipofectamine 2000 (Invitrogen, Carlsbad, CA) and incubated for 24 h. siRNA null controls were included. Cells were harvested by incubation in 0.25% trypsin with 0.53 mM EDTA at 37°C and then collected by pipetting. Cells in each group were pooled and then aliquoted for: 1) total RNA isolation in RNAProtect cell reagent (Qiagen, Valencia, CA), 2) paraffin embedding by fixation in 10% neutral buffered formalin (NBF) for 24 h, 3) western blotting by pelleting and lysing as described below, and 4) mass spectrometry by pelleting by centrifugation.

### qRT-PCR

Total RNA was isolated from harvested H441 cells using the RNeasy Protect Mini Kit (Qiagen) following the manufacturer's protocol. First-strand cDNA was generated using the Ambion RETROscript kit (Life Technologies, Grand Island, NY) according to the protocol supplied by the manufacturer using 1.5 µg of each total RNA isolate and random decamers. Real-time PCR analysis was performed on each cDNA sample in a 50 µL reaction using TaqMan Universal PCR Mix and the following TaqMan Gene Expression Assays (Applied Biosystems): VEGFR2/KDR (Assay ID: Hs00911700_m1) and GAPDH (Hs03929097_g1). The thermal profile for each well was 2 min at 50°C, 10 min at 95°C, followed by 40 cycles of 15 s at 95°C and 1 min at 60°C. Triple technical replicates were included for each sample and used to calculate average C_t_ values after application of automatic threshold. The average C_t_ for GAPDH was subtracted from the average C_t_ for VEGFR2. Values were calibrated to siRNA null samples. Antilog values were graphed.

### Mass Spectrometry

H441 cell lysate on harvested cells (∼2.6×10^6^) was prepared by adding 1 mL of ice-cold lysis buffer (50 mM HEPES, 150 mM NaCl, 1% Triton X-100, 5 mM EDTA, 5 mM EGTA, 20 mM NaF, 30 mM Na_4_P_2_O_7_) containing 1× Halt Protease and Phosphatase Inhibitors (Thermo Scientific). For 3 min, cells were sonicated on ice with alternating 30 s pulse and cooling period. Lysates were clarified by centrifugation at 10,000× *g* for 10 min (4°C) and the supernatant was collected for immunoaffinity-mass spectrometry (IA-MS) analysis.

For IA-MS, rabbit monoclonal anti-VEGFR2 antibody (clone 55B11, Cell Signaling, Danvers, MA) was coupled to a Protein A/G coated 96-well microtiter plate (Thermo Scientific). The antibody was cross-linked to the protein A/G using 0.25 mM disuccinimidyl suberate (Thermo Scientific). After blocking for one hour with Blocker Casein (Thermo Scientific), lysates were diluted in buffer and loaded onto the antibody-bound plate. Following the addition of a stable-isotope labeled (SIL) peptide for use as an internal standard, mass spectrometry grade trypsin (Promega, Madison, WI) was used to carry out proteolytic digestion. Selected reaction monitoring (SRM) was used to detect the VEGFR2-specific surrogate and SIL peptides NILLSEK and NI[^13^C_6_^15^N_1_]LLSEK (Midwest BioTech; Indianapolis, IN). Protein quantitation was achieved via targeted LC-MS/MS using an AB Sciex Qtrap 5500 mass spectrometer, monitoring the *m/z* 408.7→589.3 transition.

### Western blots

Whole cell extracts were prepared by re-suspending in protease inhibitor supplemented RIPA buffer (Thermo Scientific). Samples were combined with loading buffer containing sodium dodecyl sulfate (SDS) and dithiothreitol, boiled 5 min, and then separated on NuPage 3–8% tris-acetate polyacrylamide gels (Invitrogen). Gels were transferred to a nitrocellulose membrane that were next blocked with TBS/casein for 1 h and then probed with anti-VEGFR2 antibody (55B11) overnight at 4°C. Blots were incubated with species-specific, HRP-conjugated secondary antibodies for 1 h then visualized using enhanced chemiluminescence detection (Pierce, Thermo Scientific). The anti-GAPDH primary antibody (clone 14C10, Cell Signaling Technologies) was used to verify equal protein loading on gels.

### Histotechnological preparation of cell lines

After 24 h of fixation with 10% NBF, cells were washed with PBS and the supernatant removed. The pellet was combined with Histogel warmed to 55°C (Thermo Scientific) by gentle pipetting. Solidified Histogel pellets were placed into histological cassettes and processed on an automated tissue processor using a routine schedule. Briefly, specimens were dehydrated in a series of alcohol solutions starting at 60% and completing with 100% ethanol at 38°C, cleared multiple times with xylene at 38°C, and infused with molten Paraplast XTRA paraffin (Fisher Scientific, Pittsburgh, PA) at 56°C.

### Human tissue specimens and patient characteristics

VEGFR2 expression was first evaluated using formalin-fixed paraffin embedded (FFPE) human tumor specimens obtained from commercial sources (Indivumed and Asterand). Acquisition and processing of these tissues was confirmed to be in line with rigorous human tissue acquisition protocols that ensure collection and supply of high quality human tissues for novel biomarker studies. The parameters assessed included appropriate fixation time in neutral buffered formalin (8–24 h) and limited time to fixation (maximum 30 min). The submitted diagnoses of all tumors were independently confirmed and refined by an experienced board-certified oncologic surgical pathologist (AN). Custom-designed low-density tissue microarrays were constructed by punching 1 mm cores from the optimal areas identified in each donor tumor block.

An independent cohort of NSCLC tissues from 197 primary lung cancer patients who underwent surgical resection (pneumonectomy, lobectomy, wedge resection, wedge biopsy) of their primary lung tumors at Yale-New Haven Hospital between 1995–2003 (YTMA 79-3; Yale Tissue Microarray Facility, New Haven, CT) was also evaluated for vascular and tumor cell expression of VEGFR2. This cohort has been used in previous reports [30]–[33]. Areas of carcinoma away from normal epithelium were identified and two 0.6 mm cores were sampled from separate areas of each donor archival tumor block [34]. The entire tissue microarray (TMA) was reviewed by the study pathologist for histomorphologic evidence of squamous or glandular differentiation. In order to refine histopathologic classification of non-small cell lung cancer cases into adenocarcinoma (ADC) and squamous cell carcinoma (SCC), serial sections of YTMA 79-3 were stained for a 4-marker IHC panel (TTF-1, p63, cytokeratin 7 [CK7], and cytokeratin 5/6 [CK5/6]) to confirm the pathologic diagnoses of ADC and SCC, based on a recently proposed immunopathologic diagnostic approach [35]. Briefly, the criteria used for diagnosis of SCC were the presence of intercellular bridges, keratin pearls, individual tumor cell keratinization, and diffuse immunoreactivity for p63 with or without expression of CK5/6. The diagnosis of ADC was based on the presence of obvious glandular differentiation, extracellular and/or intracytoplasmic mucin, and strong and diffuse expression of TTF-1 with or without positivity for CK7 (Table S1). Cases with equivocal results were excluded from these two histologic subsets of NSCLC. Exclusion of individual TMA cores as a result of technical failure, attrition of sampled cores, or missing clinico-pathologic inclusion criteria resulted in 118 evaluable specimens. Clinico-pathologic characteristics of these specimens are summarized in Table 1.

**Table 1:** Clinicopathological characteristics of evaluable cases in non-small cell lung carcinoma cohort (YTMA 79-3, n = 118).

Category	Subcategory	Result
Race	Caucasian	97 (82.2%)
	African-American	15 (12.7%)
	Other	3 (2.5%)
	Unknown	3 (2.5%)
Age	Years	45–83 (mean: 65.5)
Gender	Male	63 (53.4%)
	Female	55 (46.6%)
Histopathological subtype*	Squamous cell carcinoma (SCC)	25 (21.2%)
	Adenocarcinoma (ADC)	85 (72.0%)
	Adenocarcinoma, papillary	3 (2.5%)
	Adenosquamous carcinoma	3 (2.5%)
	Large cell carcinoma	2 (1.7%)
Disease Stage	Unknown	7 (5.9%)
	IA/IB	59 (50.0%)
	IIA/IIB	17 (14.4%)
	IIIA/IIIB	27 (22.9%)
	IV	8 (6.8%)
Vital status	Alive	49 (41.5%)
	Dead	69 (58.5%)
Survival time	Months	0.1–133 (mean: 40.9, median: 26.8)

*The various histopathologic subtypes represent the final results based on a 4-marker IHC panel (TTF-1, p63, CK5/6, CK7). Using these IHC markers the original histomorphologic diagnosis was changed in 24/118 (20.3%) of NSCLC cases.

### Immunohistochemistry and pathological interpretation

FFPE sections of the YTMA 79-3 were cut (5 microns) and lifted onto Superfrost Plus adhesion slides (Fisher Scientific). Slides were dried then baked at 60°C for approximately 14 hours prior to staining. Slides were deparaffinized and rehydrated by submersion in xylene, then sequential submersion in 100%, 95%, and 70% ethanol. Antigens were retrieved by incubation in a pressure cooker set to hold at 125°C for 0.5 min in EDTA-based buffer at pH 8.5 (EDTA Decloaker, Biocare Medical, Concord, CA), citrate-based buffer at pH 6.2 (Diva Decloaker), or Tris-based buffer at pH 9.5 (Borg Decloaker). Endogenous peroxidases were treated with EnVision Flex Peroxidase Blocking Reagent (Dako; Carpinteria, CA) for 5 min, the anti-VEGFR2 antibody (clone 55B11) was applied at 0.08 ug/mL in antibody diluent (Dako) for 1 h, EnVision FLEX/HRP (Dako) was applied for 20 min, and Substrate Working Solution (1 drop of 3, 3′-diaminobenzidine substrate [DAB] per mL of substrate buffer) was applied for 10 min. Slides were removed from the stainer and counterstained with hematoxylin on an automated linear stainer following routine processes. Slides were dehydrated by sequential submersion in 95% ethanol, 100% ethanol, and xylene, and coverslipped using Cytoseal XYL (Thermo Scientific). Reagent negative controls and isotype specific IgG were used to assess non-specific staining for each tissue (data not shown). The assay was analytically validated in a CAP-accredited, CLIA-certified laboratory (Clarient, Aliso Viejo, CA) and exhibited coefficients of variation of pathological scores for intra-run repeatability, inter-run reproducibility, and inter-observer reproducibility of less than 7% (data not shown).

Serial sections of YTMA 97-3 were stained at a CLIA-certified reference lab (Clarient) using the well-established IHC assay protocols for TTF-1 (using antibody clone 8G7G3/1), p63 (BC4A4), CK5/6 (D5/16 B4), CK7 (K72.7), and CD34 (QBEnd/10).

All immunostained slides were reviewed by an experienced immunopathologist (AN) to render an assessment of VEGFR2 expression in tumor vasculature and cells. One core was scored per patient. Different scoring approaches were examined for vessels and tumor cells. Briefly, a case was interpreted as VEGFR2 positive in tumor vasculature if endothelial cells in 10% or more of the tumor stromal blood vessels exhibited unequivocal immunoreactivity for VEGFR2. An assessment of the level of intensity of tumor cell staining (range of 0, no staining; 1+, weak staining; 2+, moderate staining; 3+, intense staining) was made objectively by the study pathologist after screening the entire area of the stained tissue section. Observations were made in the tumor cell cytoplasmic and nuclear compartments simultaneously. For each level of staining intensity, the percentage of tumor cells staining for that intensity was determined. The value of each staining level (0, 1, 2 or 3) was multiplied by the respective percentage of tumor cells at that intensity level. A total VEGFR2 H-score represents the sum of the three scores and reported on a continuous scale of 0–300 (Table S1).

Images were obtained from a Leica DM 4000 B microscope with a DFC480 digital camera at ×200 or ×400 magnification using Leica Application Suite v3.7.0. Images used to quantify staining in cell lines in this report and were obtained from high-resolution digital scans (Scanscope XT; Aperio Technologies, Vista, CA). One mm diameter cell pellets were selected in ImageScope version 10.0.36.1805 and were analyzed using the Positive Pixel Count algorithm version 9.1 which returns the number of positive (brown) pixels in the selected areas in proportion to the total number of pixels where tissue is present.

### Pre-absorption assays

For initial selectivity assays, vector constructs were created to produce N-terminal HIS-tagged recombinant proteins for VEGFR1 (NP_002010.2, a.a. 799–1338), VEGFR2 (NP_002244.1, a.a. 806–1356), and VEGFR3 (NP_002011.2, a.a. 817–1363). Proteins were expressed in a baculovirus system and purified on Ni-NTA affinity (Qiagen) and Mono Q ion exchange chromatography columns. For epitope mapping studies, a series of *de novo* recombinant peptides was designed with identity to the c-terminal 150 residues of VEGFR2. The peptides were 21 or 22 residues in length and shared overlapping sequences with neighbor peptides. Peptide sequences are listed in Table S2. When performing prebsorption assays, the diluted anti-VEGFR2 antibody was combined with a 200-fold molar excess of recombinant proteins or recombinant peptides in antibody diluent (Dako). Mixtures were incubated overnight (12–15 h) at 4°C with rocking before application to immunohistochemistry.

### Statistical methods for analysis of clinical tissue data

Associations were assessed among combinations of VEGFR2 expression (vascular or tumor cell), histology, and clinical stage. For analyses within histology, only histologically pure ADC and SCC were considered. For analysis involving stage, disease stage at presentation was converted into a binary variable with levels corresponding to stage I (early disease) versus stages II–IV (later stage disease); stage subgroups (A, B) were not considered. Patients without available clinical stage information were dropped from analyses involving stage. Fisher's exact test was used for associations involving binary results of vessel expression, and the Wilcoxon rank sum test was used to assess relationships involving tumor cell H-score.

Kaplan-Meier analysis was performed and associations involving time from diagnosis to death or last follow-up (overall survival) were analyzed using Cox Proportional Hazards [36]. For analyses of tumor cell expression, VEGFR2 levels were treated as binary with cut-point selection using maximal chi-square technique described by Miller and Siegmund, which adjusts the *p*-value for testing multiple cutpoints [37]. No additional multiplicity adjustments were made across analyses. The main effects and interaction (with expression) terms of known prognostic factors (age ≤65 years vs. >65 years, sex, race [white vs. non-white], clinical stage) were also tested for inclusion in the models with VEGFR2 expression, individually for each factor, if the number of patients in each subgroup in the model was greater than 5.

All statistical analyses were performed using SAS version 9.2 (SAS, Cary, NC). A *p*-value of <0.05 was considered significant.

## Results

### The fully optimized 55B11-based IHC assay is sensitive, specific, and selective for VEGFR2 protein

**Figure 1:**
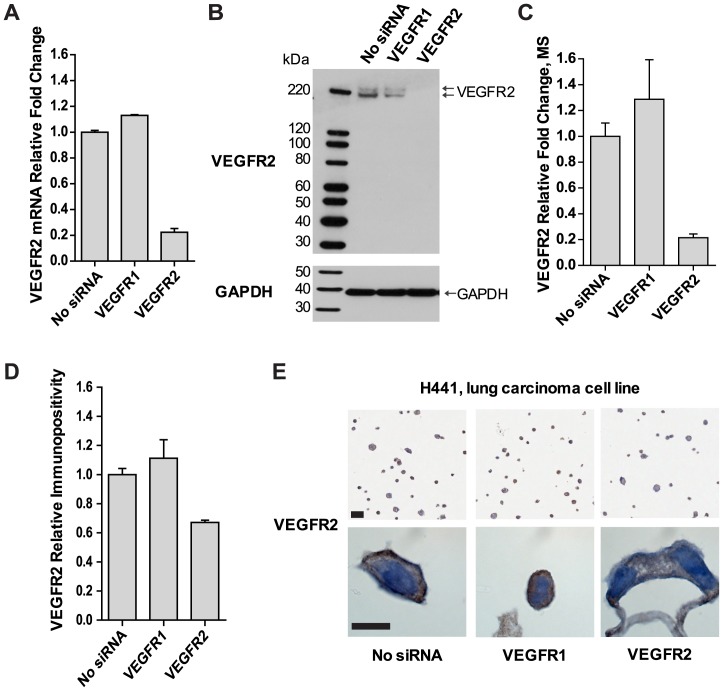
An optimized IHC assay sensitively detects VEGFR2 protein levels manipulated *in vitro*. siRNA harboring VEGFR2 sequences were used to manipulate VEGFR2 protein levels in H441 cells to inform IHC assay development. Orthogonal techniques were used to detect VEGFR2. VEGFR2 mRNA abundance was reduced 77.7% as detected by qRT-PCR analysis (A). Protein levels for VEGFR2 were determined by three methods: western blot analysis (B), IA-MS analysis (C), and positive pixel counts of immunoreactivity of IHC on FFPE cells (D). VEGFR2 protein levels were reduced by 78.5% and by 32.9% for IA-MS and IHC, respectively. VEGFR1 siRNAs were included as controls in each panel. Error bars show standard deviation of three technical replicates for panels A and C, and the standard deviation of 2 histological spots of approximately 1000 cells each for panel D. Representative VEGFR2 IHC immunoreactivity patterns exhibiting membranous and cytoplasmic immunoreactivity in trypsinized, processed, and sectioned FFPE cells (E). Original magnification, ×1000. Scale bar: 10 µm.

VEGFR2 immunoreactivity was initially characterized in the cell line H441, a lung carcinoma-derived cell line which expresses functional VEGFR2 [38], [39], in which VEGFR2-directed siRNAs were used to manipulate VEGFR2 protein levels. A selective 77.7% decrease in VEGFR2 mRNA abundance (Fig. 1A) was confirmed by 1) immunoblotting and 2) a 78.5% reduction in VEGFR2 protein levels by IA-MS (Fig. 1B, C). The above results were further substantiated by 3) a 32.9% decrease in VEGFR2 membranous and cytoplasmic immunoreactivity upon VEGFR2 knockdown (Fig. 1D, E). Taken together, these data support the specificity of 55B11 for VEGFR2 in this cell line and that our IHC assay conditions were optimized for sensitivity.

**Figure 2:**
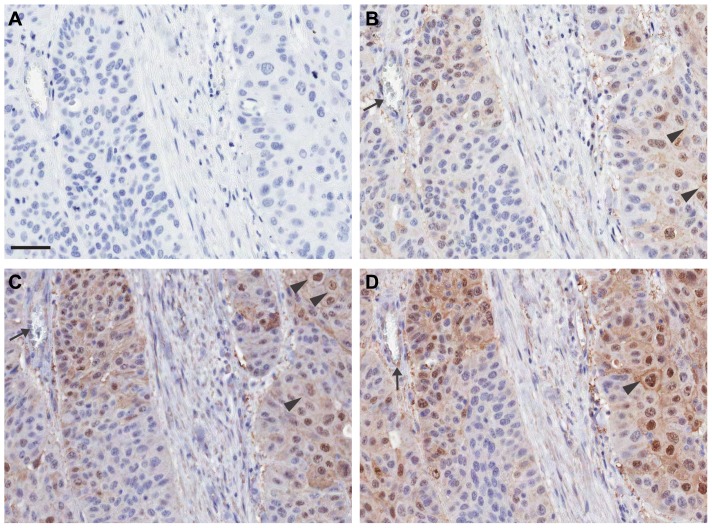
Refinement of IHC assay parameters was integral to detect subcellular immunoreactivity in tumor cells. Representative immunoreactivity is shown on serial sections of a pulmonary SCC. Slides were subjected to heat induced epitope retrieval (HIER) conditions using buffers of differing pH, followed by an optimized IHC protocol using 55B11: primary antibody reagent negative control (A), Tris buffer (B), citrate buffer (C), and EDTA buffer (D). VEGFR2 immunoreactivity in endothelial cells lining blood vessels (black arrows) and membranous (black arrowheads), cytoplasmic, and nuclear compartments of malignant tumor cells was found to be most optimal with EDTA buffer (panel D). Slides were counterstained with hematoxylin. Original magnification ×200. Scale bar: 50 µm, applicable to all panels.

The primary antibody concentration and antigen retrieval conditions were further optimized and the appropriate detection system was selected. Antigen retrieval using EDTA-based buffer at pH 8.5 was most optimal for demonstration of VEGFR2 immunoreactivity in blood vascular endothelial cells and also in different subcellular compartments (nuclei, cytoplasm, and membranes) of tumor cells in a pulmonary SCC specimen (Fig. 2).

**Figure 3:**
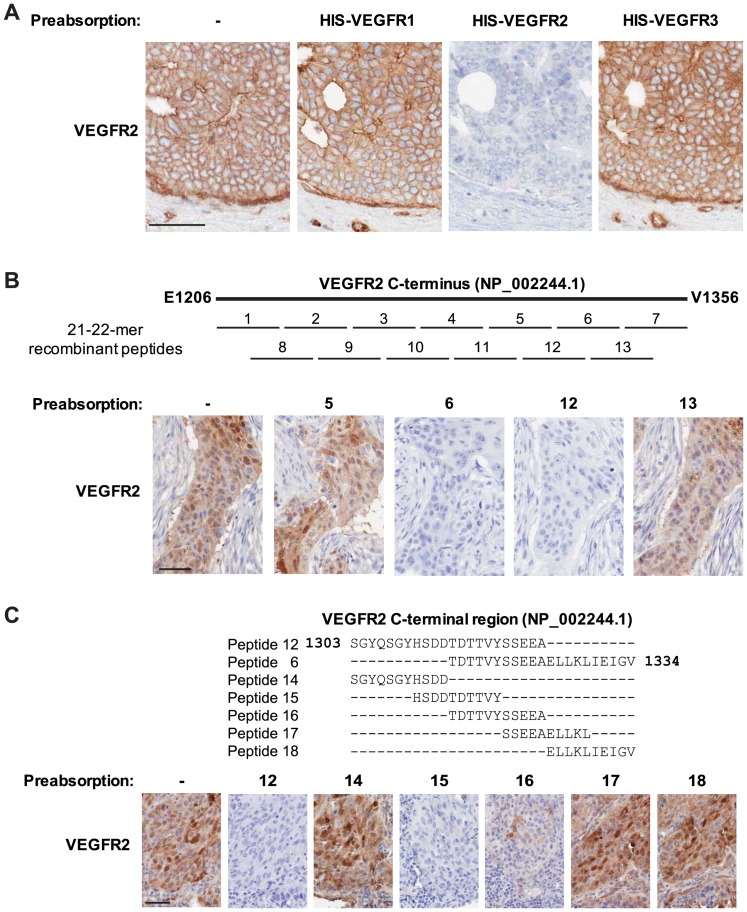
VEGFR2 IHC is specific and selective against other VEGFR family members. The anti-VEGFR2 55B11 antibody specifically recognizes a unique epitope in the sequence HSDDTDTTVYSSEEA that is harbored within the c-terminal region. (A) HIS-tagged recombinant proteins corresponding to the intracellular domains of VEGFR1, VEGFR2, and VEGFR3 were incubated at 200-fold molar excess with 55B11 prior to IHC on serial sections of ductal carcinoma of the breast. VEGFR2 and not VEGFR1 or VEGFR3 pre-absorbed the antibody leading to negative immunoreactivity. (B) Recombinant 21 or 22-mer peptides corresponding to contiguous stretches of the immunogenic sequence of 55B11 were generated as indicated in the schematic (Table S2). Each peptide was incubated at 200-fold molar excess with 55B11 prior to IHC on serial sections of pulmonary SCC. Pre-incubation with peptides 6 and 12 abolished immunoreactivity, while pre-incubation with all other peptides had no effect. The results shown for peptides 5 and 13 are representative of peptides 1–5, 7–11, and 13. (C) Recombinant 10 or 11-mer peptides corresponding to contiguous stretches of the consensus sequence of peptides 6 and 12 were generated as indicated in the schematic. Each peptide was incubated at 200-fold molar excess with 55B11 prior to IHC on serial sections of pulmonary SCC, a unique patient sample than in panel B. Pre-incubation with peptides 6 (data not shown), 12 and 15 abolished all immunoreactivity, while pre-incubation with peptides 14, 17 and 18 had no effect. Pre-incubation with peptide 16 showed an attenuated effect. All slides were counterstained with hematoxylin. Original magnification, ×200. Scale bars: 50 µm.

Because different members of the VEGF receptor family can stimulate unique patterns of cellular responses, we aimed to determine the selectivity of the VEGFR2 IHC assay. Pre-absorption of 55B11 with a VEGFR2 recombinant protein abolished specific immunoreactivity in tumor cells. VEGFR1 and VEGFR3 did not block the immunoreactivity, suggesting selectivity of 55B11 for VEGFR2 against VEGFR1 or VEGFR3 (Fig. 3A). A series of pre-absorption experiments were also used to map the epitope region of 55B11 using recombinant peptides corresponding to the c-terminal immunogenic sequences. The epitope was shown to be harbored within the amino acid sequence HSDDTDTTVYSSEEA (Fig. 3B, C). A BLAST search for a subset of this sequence that completely absorbs 55B11 (HSDDTDTTVY, Fig. 3C) showed no complete identity to any other protein, but returned limited matches to contiguous stretches of no more than 6 amino acids (e.g., growth hormone-releasing hormone receptor, low-density lipoprotein receptor-related protein 12 isoform b). However in subsequent peptide preabsorption experiments, these matching stretches of amino acids failed to abolish VEGFR2 immunoreactivity (data not shown) further substantiating the specificity of antibody clone 55B11 for VEGFR2.

### Specific VEGFR2 immunoreactivity on vascular endothelial and tumor cells using multi-tumor screening tissue microarrays

**Figure 4:**
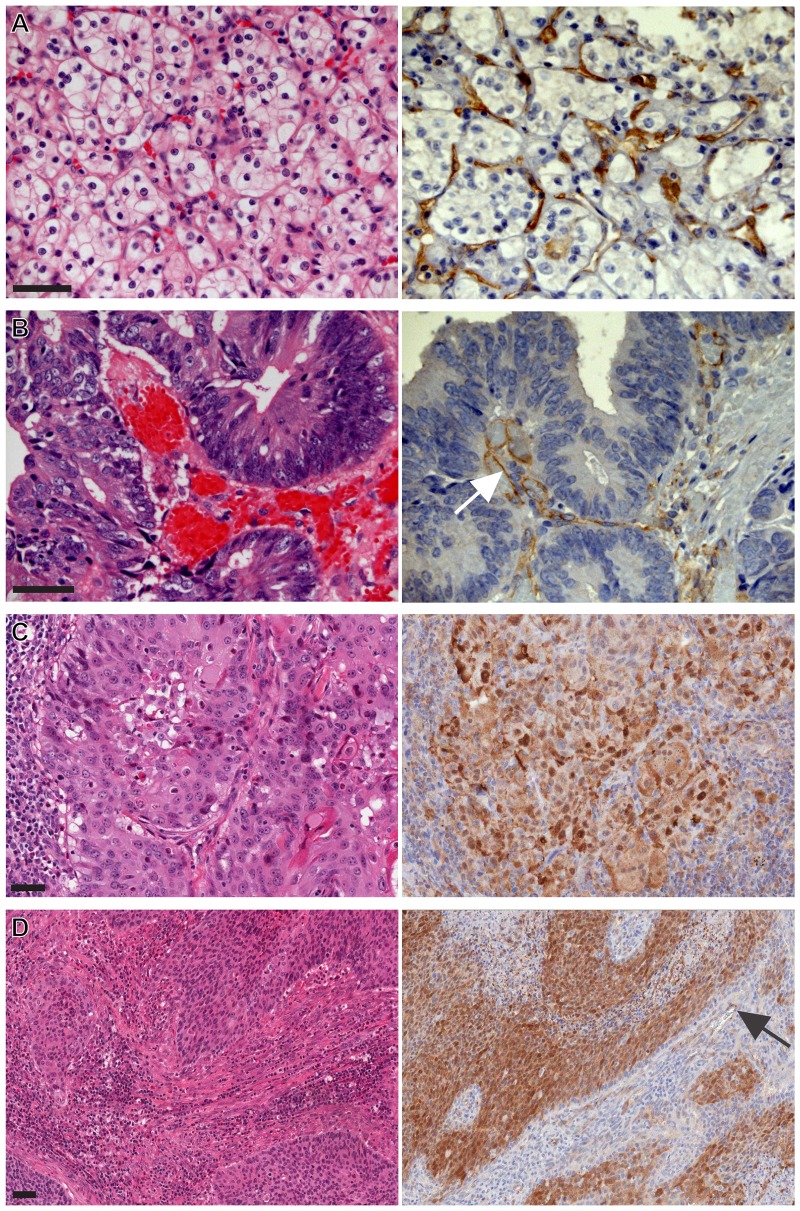
Vascular endothelial cell and tumor cell-derived VEGFR2 immunoreactivity on representative cases on a multi-tumor survey. Left panels, H? right panels, VEGFR2 IHC. (A) VEGFR2 IHC on renal cell carcinoma of the kidney showed endothelial cell immunoreactivity (×400). (B) VEGFR2 IHC on ADC of the colon showed endothelial cell immunoreactivity in the stromal mucosa. Tumor cells were negative for VEGFR2 (×400). (C) VEGFR2 IHC on SCC of the lung showed endothelial cell and a range of tumor cell-derived nuclear, cytoplasmic, and membranous immunoreactivity (×200). (D) VEGFR2 IHC showed vascular endothelial cell immunoreactivity and a range of tumor cell cytoplasmic and nuclear immunoreactivity on SCC of the cervix (×200). Immunoreactivity in endothelial cells lining vessels (white and black arrows). Slides were counterstained with hematoxylin. Scale bars: 50 µm.

To begin to assess the prevalence of VEGFR2 in various tumor tissues, a custom multi-tumor tissue microarray was constructed and stained. VEGFR2 immunoreactivity was observed in the cytoplasm of vascular endothelial cells in the majority (46/50 [92.0%]) of tumors analyzed (Table 2; Fig 4A, B). CD34-stained adjacent sections were evaluated to confirm localization of VEGFR2 in vascular endothelial cells (data not shown). Tumor cell expression of VEGFR2 was observed in 21/50 (42.0%) malignancies including carcinomas of the lung, breast, cervix, larynx, pancreas, and ovary (Table 2; Fig. 4C, D). In this screening cohort, cases of human renal cell carcinoma, hepatocellular carcinoma, pulmonary adenocarcinoma (ADC), pulmonary large cell carcinoma, and glioblastoma multiforme were negative for tumor cell expression (Table 2). Overall, a trend toward more frequent tumor cell expression of VEGFR2 was noted in squamous cell carcinomas (SCC) of the lung, cervix, and larynx. In these tumor types a pattern of immunoreactivity in the tumor cell nuclei and cytoplasm was observed, and most often it was present in both subcellular compartments within a particular case.

**Table 2:** Histopathological subtypes and VEGFR2 IHC immunoreactivity in a multi-tissue screening TMA cohort, n = 50.

Organ	Diagnosis	n	VEGFR2 vessel pos	VEGFR2 tumor cell pos
Brain	Glioblastoma multiforme	2	2	0
Breast	All types	11	10	10
	*Ductal carcinoma*	8	8	8
	*Lobular carcinoma*	3	2	2
Cervix	Squamous cell carcinoma	2	1	2
Colon	Adenocarcinoma	5	5	1
Kidney	Renal cell carcinoma	4	4	0
Larynx	Squamous cell carcinoma	4	4	3
Liver	Hepatocellular carcinoma	5	5	0
Lung	All types	8	8	2
	*Adenocarcinoma*	3	3	0
	*Squamous cell carcinoma*	3	3	2
	*Large cell carcinoma*	2	2	0
Ovary	Serous carcinoma, ductal adenocarcinoma, and clear cell type	5	4	1
Pancreas	Ductal adenocarcinoma or mucinous adenocarcinoma	4	3	2

Pos, immunopositivity.

**Figure 5:**
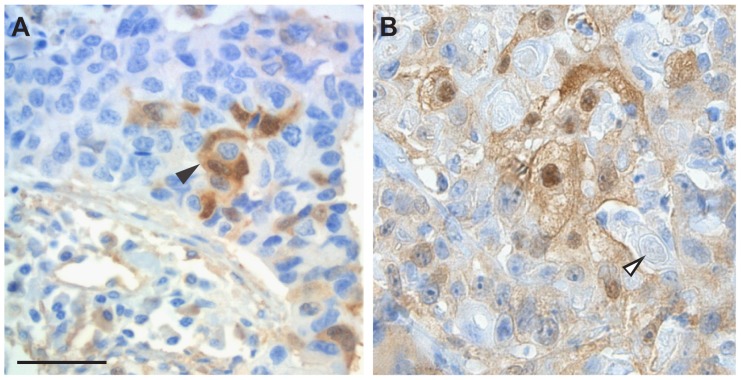
VEGFR2 immunoreactivity in areas of squamous differentiation. (A) Tumor cell-derived cytoplasmic and nuclear immunoreactivity (black arrowhead) in a focus of squamous differentiation on a background of pulmonary ADC. (B) tumor cell-derived cytoplasmic and nuclear immunopositivity in SCC of the cervix. Keratin pearls (open arrowhead) are not immunoreactive. Slides were counterstained with hematoxylin. Original magnification ×200. Scale bar: 50 µm, applicable to both panels.

Distinct morphologically-specific immunoreactivity for VEGFR2 was also identified in focal areas of squamous differentiation in otherwise typical pulmonary ADC (Fig. 5A). Interestingly, extra-cellular whorled masses of keratin were found to be VEGFR2 negative, confirming that VEGFR2 immunoreactivity in differentiated squamous tumor cells was not merely cross-reactivity to keratin (Fig. 5B). This interpretation was further substantiated by the observation that specific VEGFR2 immunoreactivity was also present in the nuclear compartment of SCC cells that typically do not contain cytoskeletal proteins like keratin (Fig. 4C, D).

Of the NSCLC specimens in this exploratory cohort, 8/8 (100%) expressed VEGFR2 in the vasculature, and 2/8 (25.0%) in tumor cells – both of which were SCC. These observations led us to further evaluate the prevalence of VEGFR2 expression in NSCLC.

### A four-marker immunohistochemical panel refines histomorphological diagnosis of NSCLC

We combined thorough histomorphologic assessment of NSCLC tissues with a panel of four IHC markers (TTF-1, p63, CK5/6, and CK7) to provide the most reliable subtyping of the NSCLC tissues analyzed (Table S1). Using this approach, the original histomorphologic diagnosis was confirmed in the majority of cases, while in 24/118 (20.3%) there was a change in the final diagnosis based on the results of this comprehensive IHC panel. The final histopathologic subtypes are represented in Table 1.

### High tumor cell expression of VEGFR2 is an adverse prognostic factor in patients with squamous cell carcinoma of the lung

**Figure 6:**
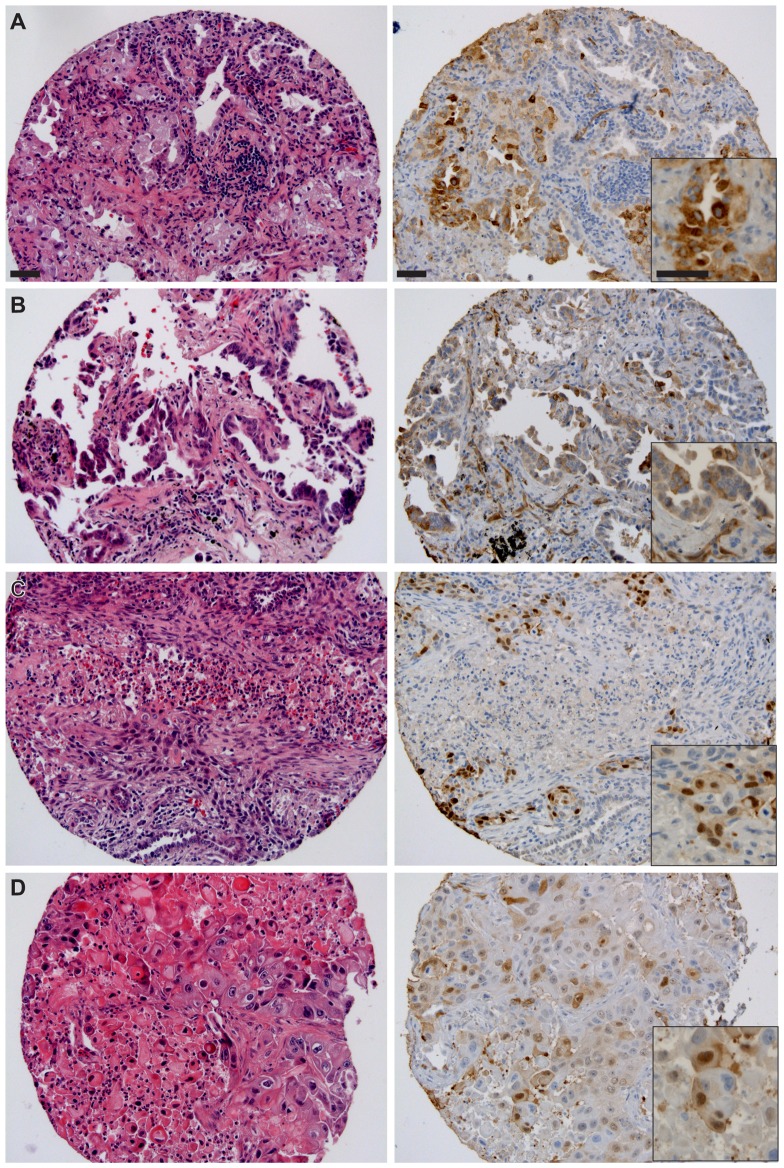
Vascular endothelial cell and tumor cell-derived VEGFR2 immunoreactivity on representative cases on NSCLC cohort YTMA79-3. Left panels, H&E and right panels, corresponding VEGFR2 IHC. (A) Representative ADC patient. Inset highlights range of cytoplasmic immunoreactivity. (B) ADC, second representative patient. Inset, note endothelial cell immunoreactivity in proximity to membranous tumor cell immunoreactivity. (C, D) Two representative SCC patients. Slides were counterstained with hematoxylin. Original magnification was ×200 for large images and ×400 for insets. Scale bars in A: 50 µm, applicable to subsequent panels.

The observation of frequent endothelial cell VEGFR2 immunoreactivity and differences in immunoreactivity of VEGFR2 in tumor cells between pulmonary ADC and SCC during our multi-tumor screening analyses led us to further investigate VEGFR2 expression on a larger, independent cohort of NSCLC tissues. A total of 118 NSCLC tissue samples were scored for VEGFR2 expression, including ADC (n = 85) and SCC (n = 25). Representative histo-morphologic and immunopathologic findings are illustrated for ADC (Fig. 6A, B) and for SCC (Fig. 6C, D).

**Figure 7:**
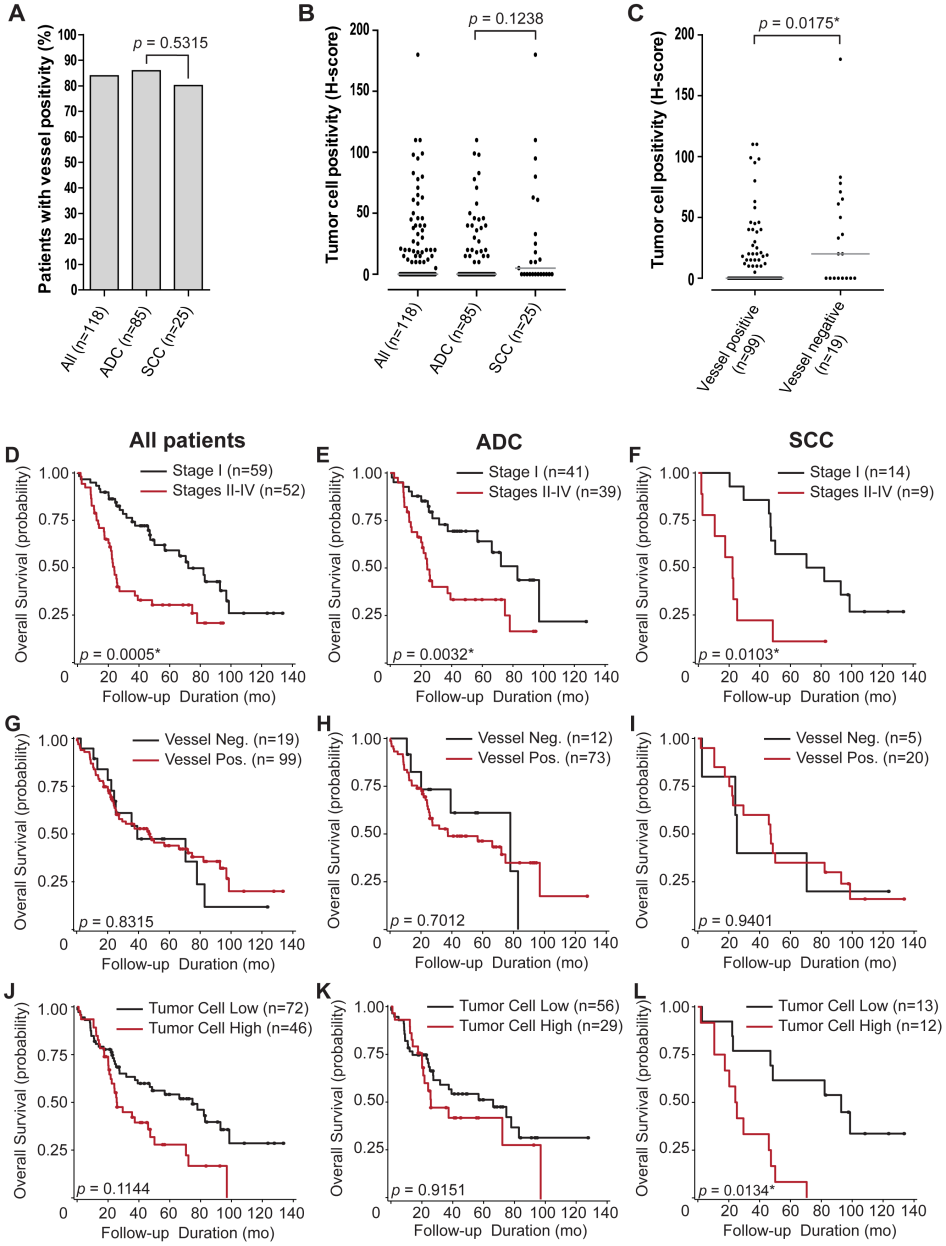
Characterization of VEGFR2 pathological scores in NSCLC patient cohort. (A) percent of patients with VEGFR2 vessel positivity per histological subtype. (B) VEGFR2 tumor cell positivity per histological subtype. (C) VEGFR2 tumor cell positivity per vessel immunoreactivity status. Light grey lines show median values for each group. *p*-values for associations are shown with statistically significant observations indicated with an asterisk. Kaplan-Meier survival curves were grouped by disease stage I or stages II–IV for: whole patient population (D), ADC (E), and SCC (F). Survival curves were also grouped by vascular endothelial cell expression of VEGFR2 for: whole patient population (G), ADC (H), and SCC (I). Additional survival curves were grouped by tumor cell-derived VEGFR2 immunoreactivity for: whole patient population (J), ADC (K), and SCC (L). Statistically optimal VEGFR2 expression cutpoints for dividing patients into groups based on survival were identified as H-scores of 10, 0, and 10, respectively. *p*-values for each curve are shown with statistically significant observations indicated with an asterisk. Points on the curves represent censored data (patients alive at follow-up time). For analysis involving VEGFR2 expression, only tumor-cell derived VEGFR2 immunopositivity in SCC patients showed a statistically significant associate (*p* = 0.0134) in median overall survival between VEGFR2 high (24.7 mo) and VEGFR2 low populations (92.8 mo).

Overall, 99/118 (83.9%) NSCLC samples were positive for vascular endothelial cell VEGFR2 staining, while 73/85 (85.9%) and 20/25 (80.0%) cases were VEGFR2-positive among the ADC and SCC subsets respectively (Fig. 7A). The difference between vascular positivity for VEGFR2 in ADC and SCC was not statistically significant (*p* = 0.5315).

VEGFR2 tumor cell staining was also observed in varying proportions of both immunohistochemically characterized ADC and SCC specimens and also among the remaining NSCLC cases including papillary ADC and mixed ADC-SCC (Fig. 7B, Table S1). Out of 118 cases, 46 (39.0%) showed tumor cell immunopositivity (H-score ≥10). The median H-score (1Q, 3Q) across all samples was 0 (0, 20). For ADC and SCC populations, 29/85 (34.1%) and 12/25 (48.0%) of the cases showed immunopositivity in tumor cells respectively, and the median H-scores (1Q, 3Q) were 0 (0, 19) and 5 (0, 33) respectively. The VEGFR2 expression scores in the tumor cells in ADC and SCC were not statistically different (*p* = 0.1238), although the trend for higher incidence of tumor cell immunoreactivity in SCC is in line with our initial observations in the multi-tumor screening cohort that comprised SCCs of various organs, including lung (Table 2).

The tumor cell H-score median for cases that were also positive for VEGFR2 in the vasculature was 0 (0, 18) and was 20 (0, 65) for cases that were also negative for VEGFR2 in the vasculature (Fig. 7C). This difference was statistically significant (*p* = 0.0175), and shows an inverse correlation between the expression of VEGFR2 in the tumor cells and the vasculature.

A significant relationship was found between patient tumor stage (Stage I vs. Stages II–IV) and overall patient survival for the entire NSCLC cohort, as well as for immunopathologically defined ADC and SCC subsets (Fig. 7D–F). These data confirmed the expected relationships between clinical tumor stage and clinical outcome in patients with NSCLC. Disease stage was also significant in some of the models which assessed the relationship between survival and VEGFR2 expression and was retained in the association models as a demographic covariate. However, age, sex, and race were not significant and were not retained. No significant association was found between vascular endothelial cell positivity and overall survival for all patients combined, or ADC, or SCC individually (Fig. 7G–I).

No significant association was found between tumor cell H-score and overall survival in analyses of all NSCLC tissues or in the ADC subset (Fig. 7J, K). The median overall survival for SCC patients with high tumor cell VEGFR2 expression was 24.7 months, and for those with a low tumor cell VEGFR2 expression (H-score <10), was 92.8 months (3.7-fold difference, n = 25, Fig 7L). This association between VEGFR2 expression in tumor cells with overall survival for SCC was significant, even after adjusting for multiple potential H-score cutpoints (*p* = 0.0134). For the analyses in the SCC subset, there was no censoring (patients alive at follow-up time) in the high tumor cell VEGFR2 expression group, and the censoring rate was 38.5% (5/13) in the low expression group. Therefore, some of the patients in the low expression group likely survived longer than was captured in the data set, suggesting a further increase in the observed survival differences between the low and high tumor cell VEGFR2 expression groups.

## Discussion

We have developed an analytically robust IHC assay for determination of VEGFR2 expression on archival human tumor tissues using a technically sound assay development and optimization approach that included high quality human tissues, confirmation of assay specificity and selectivity, stringent quality control, and analytical validation. A custom-designed TMA technology and comprehensive immuno-pathologic interpretation allowed systematic optimization of the assay parameters for optimal performance on intended use archival human tumor tissues. This comprehensive approach, similar to previously reported approaches [40], enabled us to reliably and reproducibly detect VEGFR2 immunoreactivity in stromal blood vessels in the vast majority of NSCLC cases analyzed (Table 2, Fig. 7A). Immunoreactivity for VEGFR2 was localized to the cytoplasm of the endothelial cells but was also present in tumor cells from several histologic subtypes of human malignancies.

During human cancer tissue screening with the VEGFR2 IHC assay, we characterized the subcellular expression of VEGFR2 in tumor cell membranes, cytoplasm, and nuclei from carcinomas of the lung, breast, cervix, colon, kidney, larynx, ovary, and pancreas. Although VEGFR2 is a membrane-associated tyrosine kinase receptor, previous studies have reported that VEGFR2 can dynamically internalize and translocate to the cytoplasmic and nuclear compartments [41]–[43]. Subcellular localization of phosphorylated VEGFR2 in membranous, cytoplasmic, and/or nuclear compartments of ovarian cancer and endothelial cell lines, and breast and colon tumor tissue has also been reported. These findings are consistent with the hypothesis that intracellular trafficking of VEGFR2 is linked to pathway activation – an event that may contribute to increased angiogenic response [43]–[45]. Studies in hematopoietic stem cells from mice suggest that the VEGFR2 pathway can be activated by a VEGF ligand that is not accessible to the extracellular compartment. This postulated VEGF-dependent internal or “private” autocrine loop may provide a growth advantage to neoplastic cells [46]–[48]. In addition to hematological malignancies, internal autocrine loops have been reported in solid tumors [49]. Further studies of intracellular VEGFR2 may reveal involvement of an internal autocrine loop in human tumors, thus providing a potential functional role for the intracellular VEGFR2 we observe using IHC.

The findings reported in these studies and our experience in the present study are in contrast to previous studies that characterized VEGFR2 expression using the same antibody (clone 55B11), in which little or no VEGFR2 expression was demonstrated in tumor cells from a variety of human tumor tissues analyzed [29], [50]. This includes a recent report of an analysis of more than 400 tumor tissues from various organs that showed localization of VEGFR2 primarily to tumor vasculature [29]. We attribute our ability to detect the full range of tumor cell expression of VEGFR2, in addition to vascular endothelial expression, to the optimally sensitive and specific IHC assay in combination with analysis of appropriately characterized tissue specimens. This assay has shown abundant and reproducible expression of VEGFR2 both in the tumor cell nuclei and cytoplasm in both major histologic subtypes of pulmonary carcinomas (Fig. 6).

We validated the initial immuno-pathologic observations made in a multi-tumor screening TMA and also showed the adverse prognostic significance of VEGFR2 expression in the histological subset of SCC in a well characterized cohort of NSCLC. Our results showed a statistically significant median survival difference in which pulmonary SCC patients with low expression of VEGFR2 in tumor cells survived approximately 3.7 times as long as patients with high expression of VEGFR2 in tumor cells. This study is the first to elucidate the adverse prognostic value of VEGFR2 expression in patients with immunopathologically proven pulmonary SCCs that were confirmed by a 4-marker IHC panel (TTF-1, p63, CK5/6, CK7). This IHC panel has been shown in a recent study to have utility in accurately classifying poorly differentiated NSCLC tissues on small biopsies [35]. Adding this panel to conventional histomorphologic findings allows for a definitive and reliable classification of NSCLC cases into squamous cell and adenocarcinoma. We utilized this level of stringency in classification of NSCLC cases since a high degree of accuracy and precision in lung cancer classification is becoming a more important diagnostic need in the era of emerging targeted therapeutics.

Previous studies have also shown evidence of prognostic value of VEGFR2 expression in tumor cells, vasculature, and stroma in NSCLC [20], [22], [51], [52]. Using an antibody against VEGF/KDR complex (clone 11B5), one study reported VEGF/KDR-activated microvessel density as an important prognostic factor in NSCLC and that intense VEGF/KDR angiogenic pathway activation was associated with poor post-operative outcome in more than 50% of NSCLC cases [20]. Using a different monoclonal antibody (clone A-3; Santa Cruz, Santa Cruz, CA), an independent study showed that combined high expression of VEGF, VEGFR1, and VEGFR2 proteins is associated with lower risk of progression in early pulmonary SCC. The favorable prognostic data was validated in two large independent patient cohorts [52]. However, it is not clear from their data to what extent their prognostic results can be attributed to tumor cell expression of VEGFR2. Also, it is difficult to determine the sensitivity and specificity of the primary polyclonal VEGFR2 A-3 antibody used in this and other studies [29], [52], [53]. Due to variations in antibody selection, specificity data, degree of immunopathological characterization of NSCLC tissues, and varied sub-cellular localization of VEGFR2 in these studies, it is imperative to validate the pattern and distribution of VEGFR2 in NSCLC tumor cells and its prognostic significance in larger studies using reliable assays on well characterized tumor tissues, as reported here.

Another study showed that overexpression of VEGFR2 in tumor cells was associated with poor outcome by classifying 48 NSCLC tissues into various histologic subtypes based on morphology, then scoring for VEGFR2 expression using a subjective two-tiered scheme (negative/weak, moderate/high) [51]. However, the prognostic relevance of high VEGFR2 expression in major histologic subtypes of NSCLC was not determined in that analysis. Despite some variations in their study design and scoring approach, their prognostic results are in line with what we have shown using a robust IHC assay supplemented by a comprehensive 4-marker IHC panel to further substantiate the reliability of histologic subtyping of NSCLC cases. With rapid advancements in newer targeted therapies for NSCLC patients and the major patient safety issues reported with antiangiogenic therapies in patients with squamous cell carcinoma of the lung, it is becoming increasingly important that anatomic pathologists make every effort to precisely subclassify NSCLCs into squamous cell and adenocarcinomas. And by adequate tumor sampling, it is also important that pathologists provide some assessment of the relative proportions of these two major histologic components of NSCLC in cases with mixed squamous and adenocarcinoma histologies so that novel tissue-based biomarker and clinical response data can be appropriately analyzed, especially in a clinical trial setting.

Conventional cytotoxic anticancer drugs have antiangiogenic effects, which could contribute to their anti-tumor efficacy through a variety of mechanisms–a topic that has been comprehensively reviewed by Kerbel, Kamen and Ferrara [54], [55]. Preclinical evidence indicates that combination of antiangiogenic agents with conventional cytotoxic agents results in additive or even synergistic anti-tumor effects [56]. Taxanes (e.g., paclitaxel, docetaxel) are microtubule-stabilizing chemotherapeutic agents commonly used in the treatment of small cell and non-small cell lung cancer. In a recent meta-analysis of several clinical studies using cisplatin or carboplatin plus a third-generation chemotherapeutic agent (docetaxel, paclitaxel, gemcitabine, or vinorelbine) [57]–[59], the optimum treatment strategy for patients with NSCLC has been proposed that emphasizes the importance of tumor histology (squamous vs. non-squamous) in addition to patient characteristics, EGFR mutation status, and disease biology [60]. In these analyses, pemetrexed and vinorelbine were associated with greater survival benefits in patients with non-squamous NSCLC, while docetaxel offered significant survival benefit also to pulmonary SCC patients.

Given that taxanes have been reported to have antiangiogenic activity by inhibiting vascular endothelial proliferation, motility and invasiveness *in vitro*, and tumor angiogenesis *in vivo* [61], combination of taxanes and antiangiogenic therapies targeting VEGF or VEGFR2 may provide superior efficacy, particularly in patients with squamous cell carcinoma of the lung. Based on the finding of higher incidence of VEGFR2 expression in pulmonary SCC cells and its adverse prognostic significance in the present study, it could be hypothesized that higher expression of VEGFR2 in tumor cells may be a predictor of the efficacy of antiangiogenic (or combination of docetaxel and antiangiogenic) therapy, especially in patients with SCC of the lung. A number of ongoing clinical trials are evaluating docetaxel with antiangiogenic therapies in various human malignancies, including carcinomas of the lung, breast and bladder. It would be valuable to test this and other patient tailoring hypotheses in tumor tissues from these trials.

Although a relative limitation of our study is the total number of NSCLC cases analyzed (n = 118), this can be addressed by evaluation of a larger series of similarly well characterized NSCLC tissues in the future. Also, for confirmation of assay specificity, we recognize that the siRNA data in this study are limited to one lung adenocarcinoma *in vitro* cell line. However, VEGFR2 knockdown detected by IHC using clone 55B11 has been reported previously in melanoma and ovarian carcinoma cells *in vitro* and in ovarian carcinoma *in vivo* [53], [62]. With this information in addition to supportive immunoblots, rigorous mass spectrometry data, and preabsorption experimentation, we are confident that we have been successful in developing and analytically validating a specific IHC assay for localization of VEGFR2 protein in archival human tissues, using one of the most specific commercially available anti-VEGFR2 monoclonal antibodies (clone 55B11). This clone has also been found to be the only one specific for VEGFR2 antibody in a panel of eight antibodies previously tested [29]. In our experience using this antibody, the adverse prognostic significance of high VEGFR2 expression in tumor cells remained statistically significant in pulmonary SCCs despite adjustments for testing of multiple cutpoints.

VEGFR2 also mediates VEGF signaling by intracellular association with related VEGF receptors VEGFR1 and neuropilin-1 [63]. Both of these receptors are expressed on vascular endothelial cells as well as in tumor cells [64]–[67]. High expression of VEGF-related analytes in tumor cells, including VEGFR1 and neuropilin-1, is associated with worse overall survival in breast adenocarcinoma patients [19]. However, in a study of pancreatic cancer patient cohort, low VEGFR1 expression is associated with worse overall survival [64]. Together with VEGFR2, the role of these receptors is not completely understood, but it is apparent that VEGF signaling has the capability for independent and potentially divergent roles in tumor cells and in vascular endothelial cells. Related studies of these receptor types (VEGFR1, VEGFR2, and VEGFR3) are underway in our laboratory on NSCLC cohorts.

VEGFR2 immunoreactivity in endothelial cells in the tumor vasculature was prevalent in all subsets of the lung cancer cohort that were analyzed, however when dichotomizing expression into VEGFR2 positive and negative groups, we found no significant differences in any analysis including association with overall survival. A study by Decaussin *et al.* did not show prognostic significance of VEGFR2 in NSCLC blood vessels, which is consistent with our data [68]. The lack of prognostic significance of vascular VEGFR2 in these initial studies, including ours, does not preclude the possibility that VEGFR2 expression in the vasculature can be a marker that predicts response to antiangiogenesis therapies. We therefore emphasize the relevance of comprehensive evaluation of vascular expression of VEGFR2 on tumor vasculature in pulmonary squamous cell and adenocarcinoma tissues.

In summary, we have developed a specific, selective, and sensitive IHC assay for VEGFR2 in archival human cancer tissues using a well characterized commercially available monoclonal antibody. We have also demonstrated its analytical performance on a variety of archival human cancer tissues by distinct localization of VEGFR2 in tumor blood vessels and in some of the tumor cells, and additionally we have shown data supporting specific staining. Our data have also provided evidence to support initial clinical utility in identifying an adverse prognostic subset among well characterized cases of pulmonary SCC. Strikingly, we have found an inverse correlation between VEGFR2 expression in NSCLC tumor cells and vasculature – a trend that, to our knowledge, has not been previously reported in human tissues. The observed inverse correlation was an interesting finding that merits further corroboration in large validation cohorts of NSCLC, which are currently underway in our laboratory. These findings have potential implications for defining optimal patient tailoring approaches for antiangiogenic therapies that are in clinical trials for NSCLC patients.

In conclusion, the development and analytical validation of robust and reliable tissue-based assays is critical for determination of VEGFR2 expression in NSCLC tissues. The IHC assay deployed by our laboratory is technically robust, feasible, scalable, and cost-effective, and it will be tested for prognostic and predictive value in randomized controlled clinical trials with antiangiogenic agents to evaluate its clinical utility. Furthermore, this assay may provide a technically feasible approach to selection of lung cancer patients in the future for effective tumor control by antiangiogenic therapies with or without docetaxel combination.

### Future Direction

Ongoing work includes analyses of other biologically relevant pathway biomarkers in similarly well characterized cohorts of human non-small lung and other cancer types, in order to define most clinically relevant patient tailoring tissue biomarker panels.

## Supporting Information

Table S1 **Immunopathological classification of NSCLC tissues, based on 4-marker panel; immunohistochemical expression of VEGFR2 in tumor vascular endothelial cells and tumor cells (NSCLC YTMA79-3).**
(DOCX)

Table S2 **The following recombinant peptides were used in preabsorption studies shown in** **Figure 3** **.**
(DOCX)
