# Supplementary material for: Tumor Cell Expression of Vascular Endothelial Growth Factor Receptor 2 Is an Adverse Prognostic Factor in Patients with Squamous Cell Carcinoma of the Lung
Source: PLoS One. 2013 Nov 14;8(11):e80292. doi: 10.1371/journal.pone.0080292 (PMC3828187; doi:10.1371/journal.pone.0080292)
Supplement: Table S1 — Immunopathological classification of NSCLC tissues, based on 4-marker panel; immunohistochemical expression of VEGFR2 in tumor vascular endothelial cells and tumor cells (NSCLC YTMA79-3). (DOCX) [file pone.0080292.s001.docx]

**Table S1.** Immunopathological classification of NSCLC tissues, based on 4-marker panel; immunohistochemical expression of VEGFR2 in tumor vascular endothelial cells and tumor cells (NSCLC YTMA79-3).

| **Unique patient ID (CPID)** | **TTF1 IHC** | **p63 IHC** | **CK5/6 IHC** | **CK7 IHC** | **Diagnosis (AN)** | **Vascular endothelium Positive (VP)/ Negative (VN)** | **VEGFR2, Cyt/Nuc, 1+ (%)** | **VEGFR2, Cyt/Nuc 2+ (%)** | **VEGFR2, Cyt/Nuc, 3+ (%)** | **Total Cyt/Nuc, H-score** |
| --- | --- | --- | --- | --- | --- | --- | --- | --- | --- | --- |
| 3840 | N | P | P | N | SCC | VP | 10 | 4 | 0 | 18 |
| 3841 | N | N | N | P | ADC | VP | 0 | 0 | 0 | 0 |
| 3844 | N | N | N | P | ADC | VP | 0 | 0 | 0 | 0 |
| 3847 | Nuc N (Cyt P) | N | N | P | ADC | VP | 0 | 0 | 0 | 0 |
| 3848 | P | N | N | P | ADC | VP | 0 | 0 | 0 | 0 |
| 3850 | P F | P F | N | P | ADC | VP | 30 | 5 | 0 | 40 |
| 3851 | N | N | N | P | ADC | VP | 0 | 0 | 0 | 0 |
| 3852 | N | N | N | P | LCUD | VP | 0 | 0 | 0 | 0 |
| 3853 | P W | N | N | P Vi | ADC* | VP | 15 | 0 | 0 | 15 |
| 3854 | P | N | N (RC P) | P | ADC | VP | 30 | 8 | 4 | 58 |
| 3856 | P | N | N | P | ADC | VP | 0 | 0 | 0 | 0 |
| 3858 | P | N | N | P | ADC | VP | 15 | 6 | 0 | 27 |
| 3860 | P | N | N | P | ADC | VP | 0 | 0 | 0 | 0 |
| 3862 | P | N | N | P | ADC | VP | 40 | 20 | 6 | 98 |
| 3863 | P W | P F | N | P | ADC | VP | 0 | 0 | 0 | 0 |
| 3866 | P W | N | P SC | P | ADC | VP | 6 | 10 | 4 | 38 |
| 3867 | P | N | N | p | ADC | VN | 50 | 12 | 3 | 83 |
| 3868 | P | P F | N | P | ADC | VP | 50 | 30 | 0 | 110 |
| 3870 | P W | N | N | P | ADC* | VP | 0 | 0 | 0 | 0 |
| 3873 | P W | N | N C | N C | ADC* | VP | 0 | 0 | 0 | 0 |
| 3875 | N | P | P | N | SCC | VP | 10 | 0 | 0 | 10 |
| 3876 | P W | N (RC P) | N | P | ADC | VP | 0 | 0 | 0 | 0 |
| 3878 | P | N | N | P | ADC* | VP | 0 | 0 | 0 | 0 |
| 3880 | N | P | P | N | SCC* | VP | 0 | 0 | 0 | 0 |
| 3885 | N | P | P | P F | SCC | VP | 0 | 0 | 0 | 0 |
| 3888 | N | N | N | P | ADC | VN | 0 | 0 | 0 | 0 |
| 3889 | N (RC P) | N | N | P Vi | ADC | VP | 0 | 0 | 0 | 0 |
| 3890 | N | N | N | P | ADC | VP | 0 | 0 | 0 | 0 |
| 3892 | P | N | N | P | ADC* | VP | 0 | 0 | 0 | 0 |
| 3893 | P W | N | N | P | ADC | VP | 40 | 25 | 3 | 99 |
| 3894 | N | P | P | N | SCC | VN | 0 | 0 | 0 | 0 |
| 3895 | P F W | N | N | P | ADC | VP | 0 | 0 | 0 | 0 |
| 3897 | N | N | N | P | ADC | VP | 0 | 0 | 0 | 0 |
| 3898 | N | N | N | P | ADC | VP | 15 | 15 | 0 | 45 |
| 3899 | N | P | P | N | SCC | VP | 5 | 0 | 0 | 5 |
| 3900 | N | P | P W | N | SCC | VP | 0 | 0 | 0 | 0 |
| 3901 | N | P | P | N | SCC | VP | 0 | 0 | 0 | 0 |
| 3902 | P | N | N | P Vi | ADC* | VP | 0 | 0 | 0 | 0 |
| 3903 | N | N | N | P | ADC | VP | 0 | 0 | 0 | 0 |
| 3905 | P | N | N | P | ADC | VP | 0 | 0 | 0 | 0 |
| 3908 | P F | P W | P | P | SCC | VP | 0 | 0 | 0 | 0 |
| 3909 | N | P | P | N | SCC | VN | 15 | 6 | 2 | 33 |
| 3911 | P | N | N | P | ADC* | VP | 20 | 0 | 0 | 20 |
| 3912 | P W | N | N | P | ADC | VN | 20 | 0 | 0 | 20 |
| 3914 | N | N | N | P | ADC | VP | 10 | 0 | 0 | 10 |
| 3915 | P | N | N | P | ADC* | VP | 30 | 5 | 0 | 40 |
| 3918 | N | P | P | P F | SCC | VP | 25 | 20 | 15 | 110 |
| 3920 | P W | N | N | P | ADC | VP | 0 | 0 | 0 | 0 |
| 3923 | N | P | P | N | SCC | VP | 10 | 0 | 0 | 10 |
| 3927 | P | P F | N | P | ADC | VN | 20 | 8 | 0 | 36 |
| 3934 | P | N | N | P | ADC | VN | 0 | 0 | 0 | 0 |
| 3938 | P F | N | N | P W | ADC* | VN | 70 | 4 | 0 | 78 |
| 3940 | P W | N | N | P | ADC | VP | 0 | 0 | 0 | 0 |
| 3941 | N | P F | P | N | SCC | VP | 8 | 2 | 0 | 12 |
| 3944 | P | N | N | P | ADC* | VP | 0 | 0 | 0 | 0 |
| 3945 | N | N | N | P | ADC | VP | 0 | 0 | 0 | 0 |
| 3946 | P W | N | N | P | ADC | VP | 0 | 0 | 0 | 0 |
| 3947 | P | N | N | P | ADC-PAPILLARY | VN | 20 | 0 | 0 | 20 |
| 3948 | P | N | N | P | ADC | VP | 0 | 0 | 0 | 0 |
| 3949 | P | N | N | P | ADC | VP | 10 | 3 | 1 | 19 |
| 3951 | P W | N | N | P | ADC | VP | 0 | 0 | 0 | 0 |
| 3954 | P | N | N | P | ADC | VN | 0 | 0 | 0 | 0 |
| 3955 | P W | N | N | P | ADC | VP | 0 | 0 | 0 | 0 |
| 3959 | P | P F | N | P | ADC* | VP | 0 | 0 | 0 | 0 |
| 3960 | N | N | P SC | P | SCC | VP | 0 | 0 | 0 | 0 |
| 3962 | P W | N | N | P | ADC | VP | 0 | 0 | 0 | 0 |
| 3963 | N | P F | P | P | SCC | VN | 0 | 0 | 0 | 0 |
| 3964 | N | N | N | P | ADC* | VP | 0 | 0 | 0 | 0 |
| 3967 | N | P | P | N | SCC | VP | 0 | 0 | 0 | 0 |
| 3969 | N | P | P | P | SCC | VP | 0 | 0 | 0 | 0 |
| 3972 | N | P | P | N | SCC | VN | 25 | 12 | 4 | 61 |
| 3973 | N | N | N | P F | LCUD | VP | 0 | 0 | 0 | 0 |
| 3974 | N | N | N (RC P) | P | ADC* | VP | 12 | 8 | 6 | 46 |
| 3975 | N | N | N | P | ADC | VN | 0 | 0 | 0 | 0 |
| 3976 | N | P | P | P | ADC-SCC | VP | 0 | 0 | 0 | 0 |
| 3977 | N | P W | N | P | ADC-SCC | VP | 20 | 0 | 0 | 20 |
| 3978 | P W | N | N | P | ADC | VP | 0 | 0 | 0 | 0 |
| 3979 | N | P | P | N | SCC | VP | 0 | 0 | 0 | 0 |
| 3982 | N | P | P | P F | SCC | VP | 25 | 10 | 6 | 63 |
| 3984 | P | P F | N | P | ADC | VP | 30 | 0 | 0 | 30 |
| 3985 | P | N | N | P | ADC | VP | 0 | 0 | 0 | 0 |
| 3986 | P | N | N | P | ADC | VN | 0 | 0 | 0 | 0 |
| 3987 | P | P F | N | P | ADC | VP | 15 | 0 | 0 | 15 |
| 3991 | P | N | P | P | ADC | VP | 0 | 0 | 0 | 0 |
| 3992 | N | N | N | P | ADC* | VP | 0 | 0 | 0 | 0 |
| 3994 | N | N | N | P DL | ADC* | VP | 10 | 4 | 0 | 18 |
| 3996 | N | P | P | N | SCC* | VP | 0 | 0 | 0 | 0 |
| 3997 | N | P F | P | N | SCC | VN | 20 | 35 | 30 | 180 |
| 4000 | P W | P F | N | P | ADC | VN | 20 | 15 | 0 | 50 |
| 4001 | P | N | N | P | ADC | VP | 10 | 0 | 0 | 10 |
| 4002 | P W | N | N | P | ADC | VP | 0 | 0 | 0 | 0 |
| 4004 | N | N | N | P F | ADC | VP | 0 | 0 | 0 | 0 |
| 4006 | ADC - P W; SCC - N; ADJ lung - P | SCC- P | SCC - P; ADC - N | ADC - P; SCC - N | ADC-SCC | VP | 15 | 3 | 0 | 21 |
| 4007 | N | N | N | N | ADC* | VP | 0 | 0 | 0 | 0 |
| 4008 | N | N | N | P Vi | ADC* | VN | 0 | 0 | 0 | 0 |
| 4010 | N | N | N | P Vi | ADC* | VP | 0 | 0 | 0 | 0 |
| 4012 | N | P | P | N | SCC | VP | 30 | 25 | 5 | 95 |
| 4014 | P W | N | N | P | ADC | VP | 0 | 0 | 0 | 0 |
| 4015 | N | N | N | P | ADC | VP | 0 | 0 | 0 | 0 |
| 4017 | N | N | N | P | ADC | VP | 0 | 0 | 0 | 0 |
| 4018 | P W | N | N | P | ADC | VP | 0 | 0 | 0 | 0 |
| 4019 | P W | N | N | P | ADC | VP | 0 | 0 | 0 | 0 |
| 4021 | N | N | N | P | ADC | VP | 0 | 0 | 0 | 0 |
| 4024 | P | N | N | P | ADC-PAPILLARY | VN | 15 | 10 | 10 | 65 |
| 4025 | P | N | P | P | ADC* | VP | 30 | 5 | 2 | 46 |
| 4026 | N | P | P | MG | SCC* | VP | 40 | 20 | 0 | 80 |
| 4027 | N | P W | N (RC P) | P F W | ADC* | VP | 0 | 0 | 0 | 0 |
| 4028 | P W | N | N | P | ADC | VP | 20 | 0 | 0 | 20 |
| 4030 | N | N | N | P | ADC-PAPILLARY | VP | 12 | 0 | 0 | 12 |
| 4032 | N | N | N | P | ADC | VP | 15 | 0 | 0 | 15 |
| 4033 | N | N | N | P | ADC | VP | 0 | 0 | 0 | 0 |
| 4035 | P | P F | N | P | ADC | VP | 5 | 5 | 0 | 15 |
| 4036 | P W | N | N | P | ADC | VN | 40 | 8 | 5 | 71 |
| 4037 | N | N | N | P | ADC | VP | 0 | 0 | 0 | 0 |
| 4038 | P | N | N | P | ADC | VP | 40 | 0 | 0 | 40 |
| 4045 | P | N | N | P | ADC | VP | 0 | 0 | 0 | 0 |
| 4046 | N | P | P | P F | SCC* | VP | 25 | 0 | 0 | 25 |
| 4051 | P | N | N | P | ADC | VP | 0 | 0 | 0 | 0 |

P, Positive

N, Negative

Nuc, Nucleus, nuclear subcellular compartment

Cyt, Cytoplasm, cytoplasmic subcellular compartment

VP, Vessels Positive for VEGFR2 immunoreactivity

VN, Vessels Negative for VEGFR2 immunoreactivity

F, Focal

SC, Scattered cells show immunoreactivity

RC, Rare /infrequent tumor cells immunoreactive

Vi, Variable immunoreactivity

DL, Dot-like immunoreactivity

W, Weak staining

ADC, Adenocarcinoma of the lung

SCC, Squamous cell carcinoma of the lung

LCUD, Large cell undifferentiated carcinoma of the lung

*Diagnosis differs from original clinico-pathological data (24/118, 20.3%)
