# Supplementary material for: Tumor Cell Expression of Vascular Endothelial Growth Factor Receptor 2 Is an Adverse Prognostic Factor in Patients with Squamous Cell Carcinoma of the Lung
Source: PLoS One. 2013 Nov 14;8(11):e80292. doi: 10.1371/journal.pone.0080292 (PMC3828187; doi:10.1371/journal.pone.0080292)
Supplement: Table S2 — The following recombinant peptides were used in preabsorption studies shown in Figure 3 . (DOCX) [file pone.0080292.s002.docx]

**Table S2.** The following recombinant peptides were used in preabsorption studies shown in Figure 3.

| **Figure 3 peptide nomenclature** | **Manufacturer* ID** | **Sequence** |
| --- | --- | --- |
| 1 | 5581 | evcdpkfhydntagisqylqns |
| 2 | 5582 | krksrpvsvktfedipleepe |
| 3 | 5583 | vkvipddnqtdsgmvlaseelk |
| 4 | 5584 | tledrtklspsfggmvpsksre |
| 5 | 5585 | svasegsnqtsgyqsgyhsdd |
| 6 | 5586 | tdttvysseeaellklieigv |
| 7 | 5587 | qtgstaqilqpdsgttlssppv |
| 8 | 5588 | ntagisqylqnskrksrpvsvk |
| 9 | 5589 | tfedipleepevkvipddnqt |
| 10 | 5590 | dsgmvlaseelktledrtklsp |
| 11 | 5591 | sfggmvpsksresvasegsnqt |
| 12 | 5592 | sgyqsgyhsddtdttvysseea |
| 13 | 5593 | ellklieigvqtgstaqilqpd |
| 14 | 5677 | sgyqsgyhsdd |
| 15 | 5713 | hsddtdttvy |
| 16 | 5678 | tdttvysseea |
| 17 | 5714 | sseeaellkl |
| 18 | 5679 | llklieigv |

*Midwest Bio-tech, Indianapolis, IN
